# Light induced expression of β-glucosidase in *Escherichia coli* with autolysis of cell

**DOI:** 10.1186/s12896-017-0402-1

**Published:** 2017-11-07

**Authors:** Fei Chang, Xianbing Zhang, Yu Pan, Youxue Lu, Wei Fang, Zemin Fang, Yazhong Xiao

**Affiliations:** 1 School of Life Sciences, Anhui University, Hefei, Anhui 230601 China; 2 Anhui Key Laboratory of Modern Biomanufacturing, Hefei, Anhui 230601 China; 3 Anhui Provincial Engineering Technology Research Center of Microorganisms and Biocatalysis, Hefei, Anhui 230601 China

**Keywords:** Light induction, β-Glucosidase, *Escherichia coli*, Autolysis, Immobilization, Cellulose

## Abstract

**Background:**

β-Glucosidase has attracted substantial attention in the scientific community because of its pivotal role in cellulose degradation, glycoside transformation and many other industrial processes. However, the tedious and costly expression and purification procedures have severely thwarted the industrial applications of β-glucosidase. Thus development of new strategies to express β-glucosidases with cost-effective and simple procedure to meet the increasing demands on enzymes for biocatalysis is of paramount importance.

**Results:**

Light activated cassette YF1/FixJ and the SRRz lysis system were successfully constructed to produce Bgl1A(A24S/F297Y), a mutant β-glucosidase tolerant to both glucose and ethanol. By optimizing the parameters for light induction, Bgl1A(A24S/F297Y) activity reached 33.22 ± 2.0 U/mL and 249.92 ± 12.25 U/mL in 250-mL flask and 3-L fermentation tank, respectively, comparable to the controls of 34.02 ± 1.96 U/mL and 322.21 ± 10.16 U/mL under similar culture conditions with IPTG induction. To further simplify the production of our target protein, the *SRRz* lysis gene cassette from bacteriophage Lambda was introduced to trigger cell autolysis. As high as 84.53 ± 6.79% and 77.21 ± 4.79% of the total β-glucosidase were released into the lysate after cell autolysis in 250 mL flasks and 3-L scale fermentation with lactose as inducer of SRRz. In order to reduce the cost of protein purification, a cellulose-binding module (CBM) from *Clostridium thermocellum* was fused into the C-terminal of Bgl1A(A24S/F297Y) and cellulose was used as an economic material to adsorb the fusion enzyme from the lysate. The yield of the fusion protein could reach 92.20 ± 2.27% after one-hour adsorption at 25 °C.

**Conclusions:**

We have developed an efficient and inexpensive way to produce β-glucosidase for potential industrial applications by using the combination of light induction, cell autolysis, and CBM purification strategy.

**Electronic supplementary material:**

The online version of this article (10.1186/s12896-017-0402-1) contains supplementary material, which is available to authorized users.

## Background

β-Glucosidases (EC 3.2.1.21) are a heterogeneous group of enzymes that hydrolyze β-1,4-glycosidic bond in disaccharides, oligosaccharides, aryl-, and alkyl *β*-glucosides, and release non-reducing terminal glucosyl residues [1, 2]. In recent years, *β*-glucosidases have attracted considerable interests because of their potential applications in a variety of biotechnological processes, such as production of ethanol from agricultural wastes [3, 4], release of aromatic compounds from flavorless glycosidic precursors [5], and synthesis of useful *β*-glucosides [1], etc. β-Glucosidases are ubiquitous in all domains of living organisms including Archaea, Eubacteria, and Eukaryotes [6]. However, production of β-glucosidases from native sources has been a great challenge due to low level enzyme expression and high costs in protein purification [2, 7].

To overcome these disadvantages, heterologous host strains have been employed to produce β-glucosidases. Among them, *Escherichia coli* has been widely used in the production of heterologous *β*-glucosidase, because it has been well characterized in terms of molecular genetics, physiology and expression systems [8]. Nonetheless, a number of bottlenecks exist when large scale production of *β*-glucosidases (from *E. coli*) is needed. As *E. coli* and T7 promoter are the most frequently used prokaryotic expression combinations, the addition of chemical inducers such as isopropyl β-D-1-thiogalactopyranoside (IPTG) at a final concentration of about 1 mM or more is needed to achieve maximal production of the desired proteins (Additional file 1: Table S3). IPTG is expensive, toxic, and difficult to remove during downstream operations [9]. Another obstacle on the production of *β*-glucosidases using *E. coli* is that they are often expressed as intracellular proteins [10–13]. Cell wall breakage is required in order to obtain the target protein. This process is costly and energy consuming because of the robust cell wall, which is consisted of lipopolysaccharides, an outer membrane, a peptidoglycan layer, and an inner membrane [14]. Furthermore, time-consuming and laborious protein purification steps using different kinds of chromatography such as Ni^2+^-NTA chromatography are necessary to purify the target protein.

Among protein induction strategies, optogenetic approaches have demonstrated that light can have precise control over cellular functions. The unique variability of the stimulus light, including wavelength and intensity, allows for specific triggering of cellular events in a non-invasive and highly resolving spatiotemporal fashion [9, 15]. Several light-sensitive protein domains have been engineered as optogenetic actuators to spatiotemporally control protein expression [16]. On the other hand, compared with traditional methods, cell breakage by autolysis is attracting much attention [17]. The *SRRz* lysis gene cassette, which comes from bacteriophage Lambda, has shown high efficiency in *E. coli* autolysis [18, 19]. Moreover, modules such as cellulose binding module (CBM) have been successfully used as fusion protein tags. They have been widely used for the immobilization of recombinant proteins for industrial purposes [20, 21].

Bgl1A(A24S/F297Y) is a double mutant of β-glucosidase Bgl1A (GenBank accession No. GU647096). It exhibits excellent ethanol and glucose tolerance, has good pH- and thermostability, and displays high hydrolysis rates for isoflavone glycosides [22]. In order to explore new strategies to express β-glucosidase in a cost-effective and simple way, we co-expressed β-glucosidase Bgl1A(A24S/F297Y) with the CBM from *Clostridium thermocellum*, using the combination of light induction, cell autolysis, and CBM purification strategy. Protein expression was induced by the light activated cassette YF1/FixJ [22, 23]. After induction, cells were autolyzed using the SRRz lysis system, and the enzyme in the lysate was purified using cellulose by a one-step centrifugation. Our results showed that the combination of light induction, cell autolysis, and CBM purification provide a promising revenue to efficient and economic production of β-glucosidase.

## Methods

### Bacterial strains, chemicals and culture media

The strains and plasmids used in this study were listed in additional file (Additional file 1: Table S1). *E. coli* Trans5α (TransGen, Beijing, China) was used for plasmid construction. *E. coli* BL21(DE3) and *E. coli* BL21(DE3)pLysS (TransGen, Beijing, China) were used as the hosts for Bgl1A(A24S/F297Y) production. Ampicillin, chloramphenicol, and IPTG were purchased from Sangon Biotech (Shanghai, China). *p*-Nitrophenyl β-D-glucopyranoside (*p*NPG) was from Sigma-Aldrich (St. Louis, MO, USA). The insoluble microcrystalline cellulose with an average particle size of 25 μm was acquired from Aladdin Chemistry (Shanghai, China). Ni^2+^-charged chelating sepharose fast flow was purchased from GE Healthcare (Uppsala, Sweden). All other chemicals were of analytical grade unless otherwise specified. Standard TB medium (per liter contains 4 g glycerol, 24 g yeast extract, 12 g peptone, 17 mM KH_2_PO_4_, and 72 mM K_2_HPO_4_) was used as culture medium in 250-mL Erlenmeyer flasks. Modified TB medium (per liter contains 15 g glycerol) was employed in high cell density culture (HCDC), the feeding solutions contained 45 g tryptone, 45 g yeast extract, and 500 g glycerol per liter.

### Construction of engineered strains

**Fig. 1:**
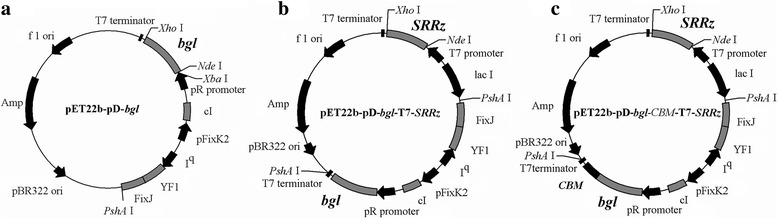
Construction of plasmids pET22b–pD-*bgl* (**a**), pET22b–pD-*bgl*-T7-*SRRz* (**b**) and pET22b–pD-*bgl-CBM*-T7-*SRRz* (**c**). The promoter region of the *Bradyrhizobium japonicum* FixK2 protein (pFixK2), promoter of the *lac* repressor gene (I^q^), lambda phage promoter (pR), lambda phage repressor protein (cI), light-sensitive histidine kinase (YF1), and regulator of pFixK2 (FixJ) were included in light induced cassette pDawn [24]

In order to evaluate the effect of light on Bgl1A(A24S/F297Y) expression, the light inducible expression plasmid pET22b–pD-*bgl* was constructed. Briefly, pET-22b vector was digested with *Xba* I and *Psh*A I to excise the T7 promoter region and lac *I* open reading frame and ligated with the similarly digested light activated cassette sequence (GenBank accession number JN579121) [24] synthesized by Sangong Biotech (Shanghai, China), resulting in vector pET22b–pD. Gene sequence of Bgl1A(A24S/F297Y) was amplified using the primer pair listed in additional file (Additional file 1: Table S2) and ligated into pET22b–pD in the *Nde* I and *Xho* I digestion sites under the control of light activated cassette, generating pET22b–pD-*bgl* (Fig. 1a). This plasmid was transformed into *E. coli* BL21(DE3) for light induction of Bgl1A(A24S/F297Y).

To combine the light induction and cell autolysis processes, *SRRz* gene from bacteriophage Lambda was cloned and inserted into *Nde* I and *Xho* I digestion sites of pET-22b under the control of T7 promoter, generating plasmid pET22b-T7-*SRRz*. Then the pD-*bgl* sequence that contains the light activated cassette and the *bgl*, cloned from pET22b–pD-*bgl*, was inserted into the *Psh*A I digestion site of pET22b-T7-*SRRz*, generating plasmid pET22b–pD-*bgl-*T7-*SRRz* (Fig. 1b)*,* which was then transformed into *E. coli* BL21(DE3)pLysS and used for protein production and cell autolysis.

In order to use cellulose as substrate for immobilization, the CBM gene from *Clostridium thermocellum*, a cellulosome-producing bacterium, was codon optimized (GenBank accession number KY994538) according to the codon preference of *E. coli* and synthesized by Sangong Biotech (Shanghai, China), and then fused to the C-terminal of Bgl1A(A24S/F297Y) with a flexible linker GSAGSA using overlap extension-PCR, generating plasmid pET22b–pD-*bgl-CBM*-T7-*SRRz* (Fig. 1c). The plasmid was transformed into *E. coli* BL21(DE3)pLysS for further research.

### Protein expression in 250-mL Erlenmeyer flasks

In order to induce Bgl1A(A24S/F297Y) expression with light, BL21(DE3)/pET-22b-pD-*bgl* was cultivated at 28 °C in 50 mL TB medium supplemented with 100 μg/mL ampicillin in an orbital shaker under dark condition. Induction was started when the cell density (*OD*_600_) reached 0.6, 3.0, and 6.0, respectively, using white light LED belt with light intensity of 6500 ± 200 lx, to investigate the influence of cell density on Bgl1A(A24S/F297Y) production. The induction was maintained for 24 h before harvest. *E. coli* BL21(DE3) containing pET22b-T7-*bgl* was used as control, and was induced by IPTG as described previously [22].

When inducing cell autolysis and releasing Bgl1A(A24S/F297Y) or Bgl1A(A24S/F297Y)-CBM from the cytoplasm, *E. coli* BL21(DE3)pLysS containing plasmid pET22b–pD-*bgl*-T7-*SRRz* or pET22b–pD-*bgl-CBM*-T7-*SRRz* were cultivated at 28 °C in 50 mL TB media supplemented with 100 μg/mL ampicillin and 34 μg/mL chloramphenicol, respectively, in an orbital shaker under dark condition until the *OD*_600_ reached 0.6. Then light induction was started. Lactose was added at a final concentration of 5 g/L after light induction for 8 h. Samples were taken every 4 h after addition of inducer and incubated in 4 °C for 4 h. The lysis efficiency was determined by analyzing both extracellular and intracellular activity of the enzyme. SRRz expression induced by IPTG at a final concentration of 1 mM was used as control.

### Protein expression in 3-L bioreactors

To compare the effect of culture condition on protein expression, cultivation was performed in a 3-L bioreactor (BioFlo 115, New Brunswick Scientific Co.). Seed culture at a final concentration of 5% (*v*/v) was inoculated into the initial culture medium (containing 100 μg/mL ampicillin) for fed-batch cultivation. The culture process was divided into two phases. The first batch phase was started with an initial glycerol concentration of 10 g/L and at 28 °C in dark condition. Immediately after the sudden increase in both dissolved oxygen (DO) and pH, constant feeding of medium was conducted at a flow rate of 12 mL/L/h. During the first phase, when cell density (*OD*_600_) of 10, 25 or 40, respectively, was reached, the second phase of induced cultivation was started. The light activated cassette was induced by a LED lamp with a light intensity of 30,000 ± 2000 lx.

For cell autolysis in HCDC, the culture was incubated in dark environment and converted to light condition when *OD*_600_ reached 10. Then lactose at a rate of 0.9 g/L/h was added into the culture after *OD*_600_ reached 30. Samples induced with IPTG at a final concentration of 1.5 mM were used as controls [25, 26]. Each sample was taken every 4 h and incubated at 4 °C for 12 h. The lysis efficiency was determined by analyzing both extracellular and intracellular activity of the enzyme. During the entire process, the pH was maintained at 7.0 by automatic addition of ammonia solution (25%, *v*/v). Antifoam was added manually when necessary. To maintain the DO level of around 30% of air saturation, the agitation speed was varied from 200 to 1000 rpm. The air flow rate was 2.5 L/min. And the inlet air was enriched with pure O_2_ when necessary.

### Immobilization of Bgl1A(A24S/F297Y)-CBM onto cellulose

The cell lysate was centrifuged at 12,000×g for 10 min. The supernatant was withdrawn and diluted to 10 U/mL of β-glucosidase activity, the pH of crude enzyme solution was adjusted to 6.5 and NaCl was supplemented at a final concentration of 200 mM. Finally, 0.5 g cellulose was added into 10 mL of crude enzyme solution. The mixture was incubated at 25 °C under mild shaking for 1 h, followed by centrifugation at 4000×g for 5 min. The cellulose bound Bgl1A(A24S/F297Y)-CBM was in sediment and was washed 3 times with Na_2_HPO_4_-citric acid buffer (50 mM, pH 6.5) to remove the unbound or loosely bound protein. The protein binding efficiency was calculated by measuring the β-glucosidase activity in supernatant and cellulose suspension. Bgl1A(A24S/F297Y) with C-terminal His_6_ purified by Ni^2+^ charged Chelating Sepharose Fast Flow (GE Healthcare, Uppsala, Sweden) was used as control.

### Determination of cell density

Cell growth was monitored during cultivation by measuring *OD*_600_ using a visible spectrophotometer (INESA, Shanghai, China). Samples were appropriately diluted with 0.9% (*w*/*v*) NaCl before determination.

### Protein activity assay and gel electrophoresis

Cells were withdrawn at different time intervals and collected by centrifugation at 12,000×g for 10 min. The pellets were washed and resuspended in Na_2_HPO_4_-citric acid buffer (50 mM, pH 6.5) and disrupted by sonication. The cell lysates were centrifuged to separate the soluble and insoluble fraction. The supernatants were used to detect the enzyme activity. Simultaneously, samples including soluble and insoluble fractions were collected and analyzed by 12% (w/v) sodium dodecyl sulfate–polyacrylamide gel electrophoresis (SDS-PAGE). In the cell autolysis section, the culture medium was also used to detect the enzyme activity and analyzed by SDS-PAGE. Protein bands were analyzed by density scanning with an imaging analysis system (Bio-Rad, USA). Protein concentration was determined by the BCA method.

β-Glucosidase activity was determined using *p*NPG as substrate. The assay mixture consisted 25 μL appropriately diluted protein sample, 475 μL 50 mM Na_2_HPO_4_-citric acid buffer (pH 6.5), and 5 mM *p*NPG. Enzyme activity was determined by monitoring absorption change at 405 nm. A unit of enzyme activity was defined as the amount of β-glucosidase required for releasing 1 μmol of pNP per minute. Reactions with heat-treated samples were used as controls. Enzyme activity was given as the averages of three separate experiments performed induplicate.

### Quantitative reverse transcription PCR (qRT-PCR) assay

The transcription of *bgl1A(A24S/F297Y)* and *SRRz* was assessed via qRT-PCR during fermentation. Briefly, total mRNA of BL21(DE3)pLysS/ pET22b–pD-*bgl1A(A24S/F297Y)*-T7-*SRRz* was extracted using the RNeasy Mini Kit (Sangon Biotech., Shanghai, China) following the manufacturer’s instructions. The cDNA was amplified through reverse transcription, with the total mRNA as the templates. Specific primer pairs (Additional file 1: Table S2) were designed using the Primer 5.0 software, and their specificities were confirmed by BLAST search against the *E. coli* BL21(DE3) genome. The 16 s rRNA gene was chosen as the control for normalization. qRT-PCR was performed in a 96-well plate in Applied Biosystems® 7500 Real Time PCR System using a SYBRs Premix Ex Taq TM II according to the manufacturer’s instructions (TaKaRa, Dalian, China). The obtained data were analyzed using the 2^-ΔΔCT^ method [27].

## Results

### Light induced expression of Bgl1A(A24S/F297Y) in *E. coli*

**Fig. 2:**
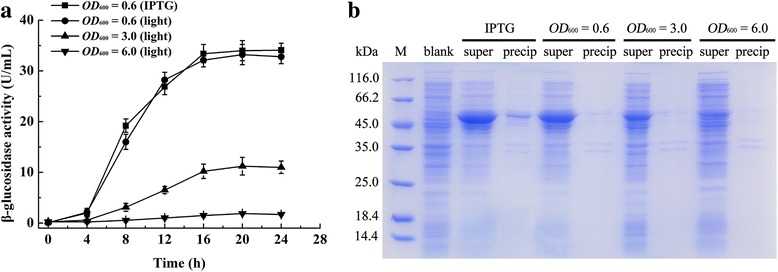
Effect of initial induction cell density on recombinant Bgl1A(A24S/F297Y) synthesis in Erlenmeyer flasks. Bgl1A(A24S/F297Y) activity after light induction at different cell densities (*OD*_600_ = 0.6, 3.0 and 6.0) and IPTG induction at *OD*_600_ = 0.6 (**a**). SDS-PAGE of Bgl1A(A24S/F297Y) induced with IPTG or light at different initial induction cell density (**b**), M is molecular marker, blank represents BL21(DE3)-pET22b without *bgl* insertion, IPTG represents BL21(DE3)/pET22b-T7-*bgl* induced with IPTG, *OD*_600_ = 0.6, *OD*_600_ = 3.0 and *OD*_600_ = 6.0 represent BL21(DE3)-pET22b–pD-*bgl* induced with light at different cell density gradients

The relationship between the yield of Bgl1A(A24S/F297Y) and cell density upon initial induction was analyzed. When cells were exposed to light at the early exponential phase (*OD*_600_ = 0.6), the activity of Bgl1A(A24S/F297Y) reached 33.22 ± 2.0 U/mL after 20 h induction (Fig. 2). However, lower Bgl1A(A24S/F297Y) activity of 11.22 ± 1.7 U/mL was obtained when the culture was exposed to light at the initial cell destiny of 3.0 (*OD*_600_). When induction is initiated at a cell destiny of 6.0, 2.87 ± 0.18 U/mL of β-glucosidase activity was obtained after identical length of induction. On the other hand, cell growth was not affected with initiation time of induction for protein expression. The expression of Bgl1A(A24S/F297Y) induced by light and IPTG were compared. Similar trends were obtained in terms of enzyme activity and cell growth, regardless of either T7 promoter or light activated cassette was used for *bgl1A(A24S/F297Y)* expression (Fig. 2). Bgl1A(A24S/F297Y) activity reached 34.02 ± 1.96 U/mL when induced with IPTG. SDS-PAGE showed that the amount of Bgl1A(A24S/F297Y) expressed corroborates with the enzyme activities (Fig. 2b).

### Autolysis of *E. coli* cells in Erlenmeyer flasks

**Fig. 3:**
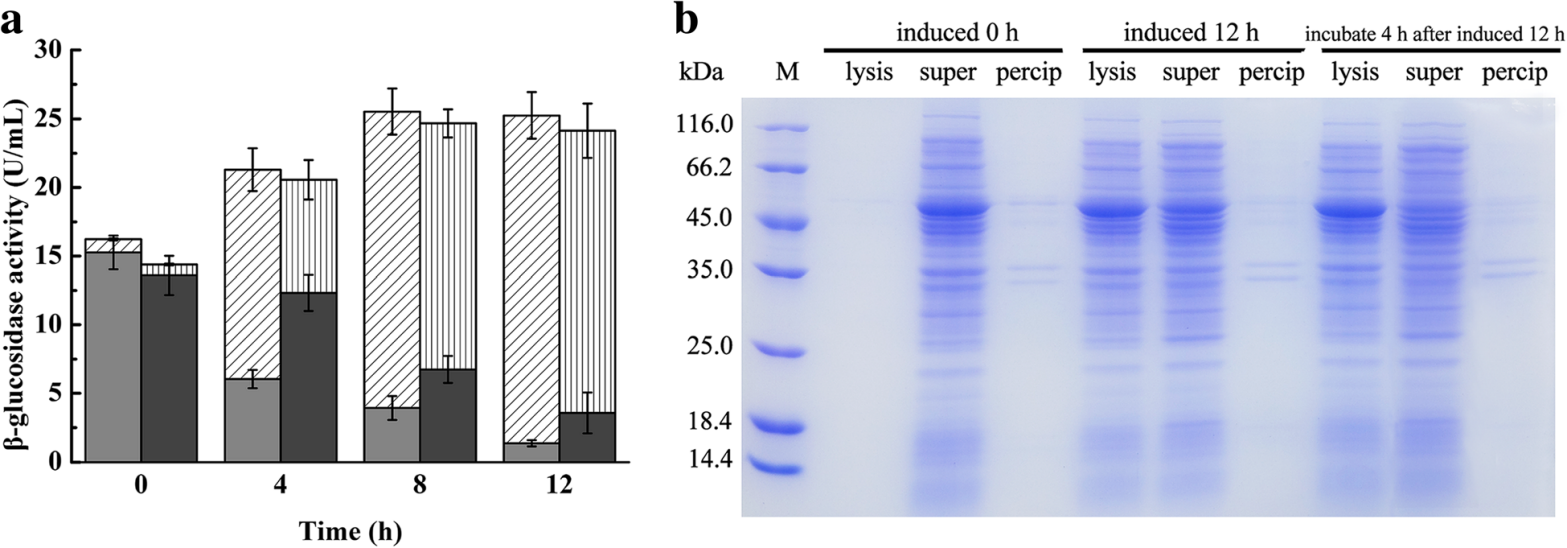
Bgl1A(A24S/F297Y) activity and SDS-PAGE assay of extracellular and intracellular fraction after cell autolysis in flasks. Slash (extracellular) and gray (intracellular) reflect β-glucosidase activity when *SRRz* lysis gene was induced with IPTG, vertical (extracellular) and dark gray (intracellular) reflect enzyme activity when autolysis was induced with lactose (**a**). The soluble and insoluble fraction of cell lysate was assayed by SDS-PAGE when autolysis was induced with lactose (**b**), M is molecular marker, lysis is supernatant of fermentation culture after cell autolysis, super and precip are supernatant and precipitate after ultrasonic fragmentation of cells that were not autolysed

In order to reduce the production cost of Bgl1A(A24S/F297Y), and to avoid protein damage by ultrasonic or chemical methods, the *SRRz* lysis gene cassette from bacteriophage Lambda was cloned into pET22b vector and under the control of T7 promoter. Furthermore, pD-*bgl1A(A24S/F297Y)* cassette was also cloned into the same vector, generating plasmid pET22b–pD-*bgl*-T7-*SRRz* (Fig. 1b). However, after it was transformed into *E. coli* BL21(DE3) and induced by light to express Bgl1A(A24S/F297Y), little protein was obtained due to cell disruption throughout fermentation caused by background expression of SRRz controlled by T7 promoter. To overcome this challenge, *E. coli* BL21(DE3)pLysS cells, in which protein expression controlled by T7 promoter was strictly regulated, was used as an alternative to express Bgl1A(A24S/F297Y) and SRRz. Lactose was used as an alternative inducer to induce *SRRz* expression. The total β-glucosidase activity reached 24.14 ± 2.14 U/mL, the lysis efficiency (extracellular enzyme activity/total enzyme activity) was 84.53 ± 6.79% after 4 h incubation (Fig. 3). As a control, when IPTG was used to induce cell autolysis, the total β-glucosidase activity reached 25.53 ± 1.64 U/mL, and the lysis efficiency was found to be 94.58 ± 3.10% (Fig. 3a). To further simplify the purification procedure, strain BL21(DE3)pLysS/pET22b–pD-*bgl-CBM-*T7-*SRRz* was used in the expression of fusion protein Bgl1A(A24S/F297Y)-CBM that contained a CBM-tag (17.4 kDa) and a GSAGSA linker at the C-terminal. The total β-glucosidase activity reached 21.45 ± 2.59 U/mL with an autolysis efficiency of 85.34 ± 5.31% when induced with lactose. Almost all of the fusion protein was expressed in soluble form (Table 1).

**Table 1:** Expression of β-glucosidase and autolysis efficiency of various strains in 250 mL Erlenmeyer flasks

strains and plasmids	inducer	β-glucosidases activity (U/mL)	autolysis efficiency
BL21(DE3) + pET22b–pD-*bgl*	Light	33.22 ± 2.01	
BL21(DE3) + pET22b-T7-*bgl*	IPTG	34.02 ± 1.96	
BL21(DE3)pLysS + pET22b–pD-*bgl*-T7-*SRRz*	Light + lactose	24.14 ± 2.14	84.53 ± 6.79%
	Light + IPTG	25.53 ± 1.64	94.58 ± 3.10%
BL21(DE3)pLysS + pET22b–pD-*bgl-CBM*-T7-*SRRz*	Light + lactose	21.45 ± 2.59	85.34 ± 5.31%

### Production of Bgl1A(A24S/F297Y) in 3-L bioreactors

**Fig. 4:**
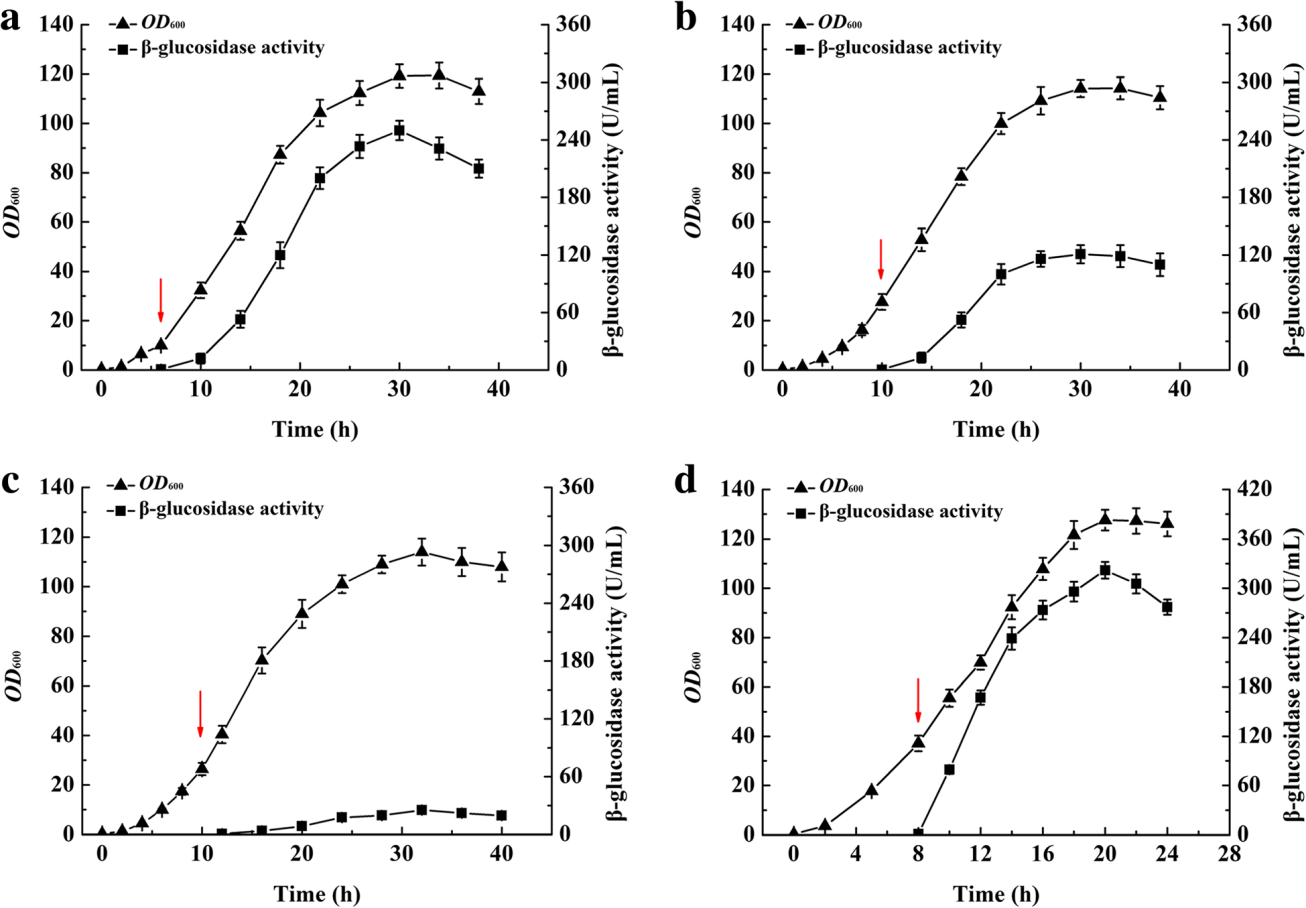
Bgl1A(A24S/F297Y) production and *E. coli* growth in fed-batch cultivations induced with light and IPTG. Arrows indicate the point of light induction at cell density of *OD*_600_ = 10 (**a**), *OD*_600_ = 25 (**b**), *OD*_600_ = 40 (**c**) and induced with IPTG at cell density of *OD*_600_ = 40 (**d**)

Light induced expression of Bgl1A(A24S/F297Y) in HCDC was investigated in 3-L bioreactors. BL21(DE3)/pET22b–pD-*bgl* was used as the expression strain. The enzyme activities reached 249.92 ± 12.25, 121.1 ± 9.87, and 25.3 ± 3.87 U/mL when induced at *OD*_600_ values of 10, 25, and 40, respectively (Fig. 4). In comparison, when expression was induced by IPTG, Bgl1A(A24S/F297Y) activity reached 322.21 ± 10.16 U/mL when induced at *OD*_600_ values of 40 (Fig. 4d). The specific activity (42.3 U/mg) of Bgl1A(A24S/F297Y) has been reported previously [22]. Based on this data, the expression of Bgl1A(A24S/F297Y) was 5.91 ± 0.29 g/L and 7.85 ± 0.24 g/L when induced with light and IPTG, respectively, in the 3-L fermentation tank.

**Fig. 5:**
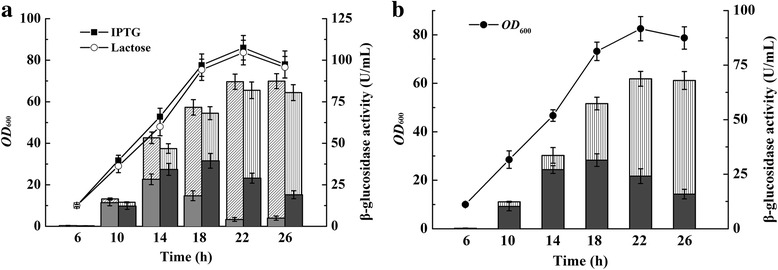
Bgl1A(A24S/F297Y) and Bgl1A(A24S/F297Y)-CBM expression in HCDC. Growth profile and activity of Bgl1A(A24S/F297Y) (**a**) and Bgl1A(A24S/F297Y)-CBM (**b**) was detected after autolysis. Slash (extracellular) and gray (intracellular) reflect Bgl1A(A24S/F297Y) activity when *SRRz* lysis gene was induced with IPTG, vertical (extracellular) and dark gray (intracellular) are enzyme activity when autolysis was induced with lactose

**Fig. 6:**
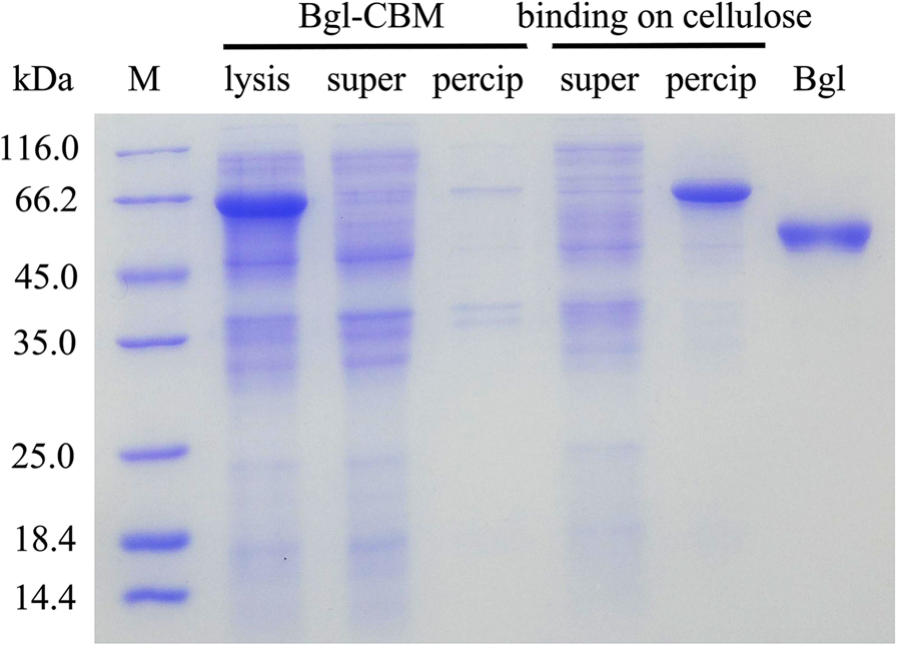
SDS-PAGE of Bgl1A(A24S/F297Y)-CBM expression in *E. coli* using light induction and adsorption onto cellulose after autolysis. M is molecular marker, lysis is supernatant of fermentation culture after cell autolysis, super and precip are supernatant and precipitate after ultrasonic fragmentation of cells that were not autolyzed. Binding on cellulose was allowed for one hour, the supernatant and precipitate were separated by centrifugation. Bgl is purified protein using hexahistidine affinity tag

When lysis efficiency was investigated in HCDC, BL21(DE3)pLysS containing pET22b–pD-*bgl*-T7-*SRRz* was inoculated into a bioreactor and cultured as described in the method section. When lactose was used as the inducer, the highest total enzyme activity of 83.88 ± 5.34 U/mL was detected, and the final lysis efficiency was 77.21 ± 4.79% after 12 h incubation (Fig. 5a). SDS-PAGE showed that most of the target protein was released into the culture after induction with lactose (Additional file 1: Figure S1). When IPTG was used as an inducer, the total enzyme activity reached the maximum value of 90.6 ± 4.35 U/mL. The final lysis efficiency was 91.78 ± 5.27%. When the fusion protein Bgl1A(A24S/F297Y)-CBM was expressed with light induction, lactose was used as an inducer in the expression of SRRz and the fusion enzyme was released into the lysate after cell autolysis (Fig. 5b). The total β-glucosidase activity reached the maximum value of 72.95 ± 4.82 U/mL during fermentation, and the final lysis efficiency was 77.21 ± 4.79% (Fig. 6).

### Immobilization of fusion protein onto cellulose

To further simplify the steps of purification and lower the cost of protein isolation, crude Bgl1A(A24S/F297Y)-CBM (supernatant after cell lysis) was adsorbed onto cellulose. The adsorption efficiency of the crude fusion protein was measured. After adsorption, the enzyme activity remained in the supernatants and adsorbed on cellulose were detected. Our results indicated that the binding between Bgl1A(A24S/F297Y)-CBM and cellulose could be accomplished within 1 h (Fig. 6). The yield of the fusion protein reached 84.6 ± 3.51% in 30 min, and reach the maximum of 92.20 ± 2.27% after 1 h adsorption. In contrast, Bgl1A(A24S/F297Y) with C-terminal His-tag had the yield of 91.1% with a Ni^2+^-NTA column (Table 2).

**Table 2:** Purification of Bgl1A(A24S/F297Y) and Bgl-CBM from *E.coli*

	Steps	Total protein (mg)	Total activity (U)	Specific activity (U/mg)	Purification (Fold)	Yield (%)
Hexahistidine affinity	Cell extract	9.23	120.62	13.07	1	100%
	Purificated	2.64	109.89	41.52	3.17	91.1%
Cellulose affinity	Cell extract	9.65	116.88	12.11	1	100%
	Purificated	3.29	105.89	32.12	2.65	90.6%

Data present was the average of three batches. Total protein = protein concentration (mg/mL) × volume (mL). Yield = total protein (mg) × purity (%)

### Gene expression analysis using qRT-PCR

**Fig. 7:**
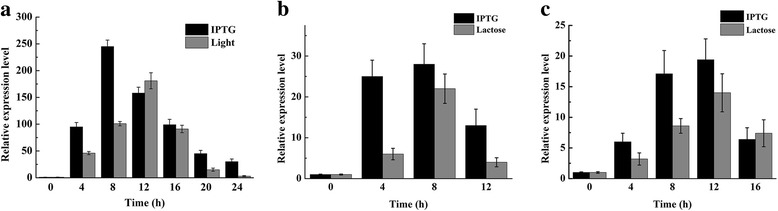
qRT-PCR analysis of *bgl* and *SRRz* in 250-mL Erlenmeyer flasks and 3-L bioreactors. The *bgl* transcription in recombinant *E. coli* BL21(DE3)-pET22b–pD-*bgl* in Erlenmeyer flasks was detected (**a**), the transcription of *SRRz* in recombinant *E. coli* BL21(DE3)-pET22b–pD-*bgl*-T7-*SRRz* was detected in Erlenmeyer flasks (**b**) and HCDC (**c**)

In Erlenmeyer flasks, qRT-PCR analysis showed that relative expression of *bgl1A(A24S/F297Y)* were 95.34 ± 9.25 and 245.22 ± 14.25 after 4 h and 8 h induction with IPTG, and decreased to 158 ± 11.44 within 12 h (Fig. 7a). In comparison, relative expressions of *bgl1A(A24S/F297Y)* were 46.54 ± 5.25 and 101.24 ± 9.25, and reached to 181.44 ± 17.35 after 12 h of light induction. In cell autolysis, qRT-PCR analysis results showed that the relative expression level of *SRRz* was 22.21 ± 3.66 at 8 h post induction with lactose as an inducer (Fig. 7b). In contrast, the relative expression level of *SRRz* was 25.12 ± 4.43 after 4 h induction with IPTG, and reached 28.32 ± 5.45 after 8 h of induction. In 3-L bioreactors, the qRT-PCR analysis showed that the relative expression level of *SRRz* was 14.04 ± 3.16 after induction with lactose for 12 h, as a control, this value increased to the maximum of 19.4 ± 3.42 after induction with IPTG for 12 h (Fig. 7c).

## Discussion

Most recombinant β-glucosidases were expressed in *E. coli* BL21(DE3) or its derivatives using T7 promoter with IPTG as inducer (Additional file 1: Table S3). Generally, proteins expressed with this method are accumulated in the intracellular portion of the cell that need to be disrupted to acquire the target protein [10, 28]. These strategies have high cost and involve tedious purification procedures. Therefore, the search for a cheaper and simple production strategy becomes important and necessary. In this study, we employed a light induced cassette in *E. coli* for the production of β-glucosidase Bgl1A(A24S/F297Y) in 250-mL Erlenmeyer flasks and 3-L bioreactors. Subsequently, lactose, a cheap and harmless material, was used to induce the SRRz cell autolysis system to lyse the cells and the CBM immobilization strategy was executed to purify the β-glucosidase without the need of column chromatography.

Light induced promoter system has been applied to study gene function, optimize metabolic pathways, and control biological systems both spatially and temporally [29]. Particularly, Ohlendorf et al. reported that light induced cassette pDawn has several advantages in expressing proteins [24]. Here we employed light induced cassette pDawn in *E. coli* to express a mutant β-glucosidase, Bgl1A(A24S/F297Y). Our results indicated that cell density at which the initial induction takes place is a key factor for Bgl1A(A24S/F297Y) production. In pDawn expression system, both YF1 and FixJ are constitutively expressed [24]. YF1 is a light-sensitive histidine kinase and phosphorylates FixJ in the absence of light. The phosphorylated FixJ drives the expression of lambda phage cI that binds to lambda phage promoter pR. Promotor pR is the key in controlling the expression of Bgl1A(A24S/F297Y). In the case of light stimulation, the expression of cI was repressed and Bgl1A(A24S/F297Y) expression was stimulated. Based on this mechanism, and the fact that light is relatively poor in penetrating tissues [30], in our research, it might also be difficult for the light to penetrate the cultivations at high cell density. Therefore, the expression of cI keeps going on, and results in the reduced production of Bgl1A(A24S/F297Y) from pR promoter.

T7 promoter has been recommended as one of the strongest promoters used to produce proteins in *E. coli*, and has been successfully used for the expression of many heterologous proteins [31, 32]. Our results showed that light induction may be used as an alternative of IPTG induction for the expression of Bgl1A(A24S/F297Y). However, the qRT-PCR results revealed that the relative expression of *bgl1A(A24S/F297Y)* were 46.54 ± 5.25 and 101.24 ± 9.25, and reached 181.44 ± 17.35 after 12 h of light induction in flasks. In comparison, the relative expression of *bgl1A(A24S/F297Y)* rose up to 245.22 ± 14.25 after an 8 h induction with IPTG. These results showed that the response time of the light activated cassette was longer than that of the T7 promoter, and the efficiency of light induction is more tempered than IPTG induction, which could slow down the expression rate of heterologous protein, and thus facilitate protein folding. In accordance with this fact, when IPTG was used as the inducer, some of Bgl1A(A24S/F297Y) end up in the form of inclusion body. But when using light as the inducer, almost all Bgl1A(A24S/F297Y) was in soluble form. Therefore, light induction may be used to produce some proteins that are difficult to express in soluble form.

Conventional cell breakage methods include mechanical, chemical, or biological strategies [33]. These methods are costly and involve cumbersome operations. For example, sonication is simple and powerful, but it is difficult to scale up, and local heating may damage or denature the target protein [34]. Enzymatic cell breakage can also be adapted on a large scale, but this method was hindered by the cost of enzymes used. Meanwhile, most β-glucosidases produced in *E. coli* exist as intracellular proteins that require cell breakage to obtain the target protein [10, 11, 13, 35, 36]. Therefore, autolysis using proteins such as SRRz has attracted much attention during recent years. For instance, the lysis efficiency of SRRz was found to be consistent and above 60% when controlled by UV-inducible promoters *recA* and *umuDC,* and measured using β-galactosidase as the reporter at 30 °C [19]. In comparison, when the *SRRz* gene cassette was inserted after heat-inducible promoter, lysis efficiency reached 97.0 ± 0.8% after heat induction at 42 °C for 30 min. However, the lysis efficiency was only 76.0% when the seed liquid was maintained at 35 °C overnight [18]. In this study, the *SRRz* gene was controlled by the strong T7 promoter in a strict expression host BL21(DE3)pLysS. The lysis efficiency reached 84.53 ± 6.79% and 94.58 ± 3.10% when lactose or IPTG was used as inducer. Compared to IPTG, the induction effect of *SRRz* by lactose is delayed, since lactose needs to be taken up by the cells (by the *lacY*-encoded lactose permease), and then metabolized to the actual inducer of allolactose [37].

To avoid background SRRz expression that will lead to cell autolysis before gene expression takes place, in this study, several strategies were employed to ensure that gene cassette was reliable during protein expression. Firstly, Bgl1A(A24S/F297Y) and SRRz were separately controlled by light induced promoter and T7 promoter, respectively. Secondly, *E. coli* BL21(DE3)pLysS was used to prevent background SRRz expression since protein expression in this strain is controlled by T7 promoter that was strictly regulated. Thirdly, the expression of β-glucosidase and cell autolysis were performed as two independent induction processes. After β-glucosidase expression reached a plateau by using the light-activated cassette YF1/FixJ [22, 23], lactose was added to trigger the expression of lysis protein SRRz. Based on these strategies, there were few proteins released into the culture medium caused by cell lysis (Fig. 3b), confirming the reliability of the gene cassette.

HCDC are commonly used in numerous manufacturing processes for large-scale production of biological product with reasonable cost. For example, the productivity of silk-elastin-like protein reached 4.3 g/L in a fed-batch culture of *E. coli* BL21(DE3) [38]. Anuradha et al. reported a final interferon-β concentration of 4.8 g/L using a modified *E. coli* strain BL21(*glpK* ^+^) [39]. Under the fed-batch conditions, the final product titer of the short peptide surfactant, DAMP4, reached 7.4 g/L in *E. coli* BL21(DE3) [40]. However, there have been limited examples referring to heterologous protein expression in *E. coli* with light induction in HCDC. Here we demonstrated that the expression of Bgl1A(A24S/F297Y) can reach up to 5.91 ± 0.29 g/L with light induction initiated at *OD*_600_ of 10, comparable to the productivities reported by other inducers. On the other hand, cell destiny in HCDC was much higher. It may be one of the bottlenecks that affects the light signal, and consequently affects the protein productivity. As a result, in HCDCs, the time-point of induction is an important factor because induction at different cell density can result in different production yields [41]. Based on these facts, the final Bgl1A(A24S/F297Y) activities observed in 3-L bioreactors were negatively correlated with the initial induction cell density. Although the light intensity was improved in 3-L bioreactors than in flasks, it was still difficult for the light to penetrate effectively into the cultivation when the *OD*_600_ was higher than 25 (Fig. 4).

Industrial processes require the efficient production of purified enzymes with simple and rapid protocols. Fusing a heterologous protein with an affinity tag has been proven to be a very useful method to purify and immobilize protein [42, 43]. A large number of affinity systems, such as the glutathione S-transferase and maltose-binding protein, have been used as affinity tags for the purification and immobilization of fusion proteins. However, most of them are costly. CBM is an attractive affinity tag for protein purification and immobilization for its highly specific binding ability, efficient release of bound protein under non-denaturing conditions, enhanced protein folding and secretion/solubility [44, 45]. These features make CBMs an ideal tool in purification and immobilization of enzymes for industrial scale applications. The CBM fusion strategies have been successfully applied in the immobilization of several enzymes such as γ-lactamase (EC 3.5.2.-) and lipase (EC 3.1.1.3) [46, 47]. A CBD fusion β-galactosidase CBD-BgaL3 was directly adsorbed onto microcrystalline cellulose with immobilization efficiency of 61% [43]. These successful examples have prompted us to fuse Bgl1A(A24S/F297Y) with CBM using the light-induction system. In this study, we preferred family 3 CBM because it possesses irreversible and strong cellulose binding capacity [44, 48] that facilitate the purification and immobilization of the fusion protein from cell lysate. The results showed that CBM used in this work effectively purified and immobilized target protein from cell lysate. We further demonstrated that the immobilized enzyme kept 92.20 ± 2.27% of the protein’s activity after one-hour adsorption at 25 °C. The yield of cellulose affinity and hexahistidine affinity were almost the same, suggesting that cellulose can serve as a convenient, efficient, and economic support in the purification and immobilization of β-glucosidase.

## Conclusions

In summary, a mutant β-glucosidase Bgl1A(A24S/F297Y), tolerant to glucose and ethanol, was produced using a high-efficiency and cost-effective strategy that combines light induction and cell autolysis in *E coli*. The yield of recombinant Bgl1A(A24S/F297Y) induced by light was comparable to that induced by IPTG in 250-mL flask. Furthermore, 84.53 ± 6.79% of the total β-glucosidase was released into the lysate after the induction of *SRRz* lysis gene cassette from bacteriophage Lambda with lactose. A cellulose-binding module (CBM) from *Clostridium thermocellum* was codon optimized and fused to the C-terminal of Bgl1A(A24S/F297Y) with a flexible linker. More than 92.20 ± 2.27% of enzymatic activity was transferred to the cellulose after one-hour adsorption at 25 °C. Our results indicated that combination of light induction and cell autolysis is an efficient and cost-effective way for large-scale production of β-glucosidase that has great potential applications in biotechnology and industry.

## Additional file

Additional file 1: Table S1. Bacterial strains and plasmids used in this study. **Table S2.** Oligo nucleotides used in this work. **Table S3.** Expression of prokaryotic β-glucosidase. **Figure S1.** SDS-PAGE of Bgl1A(A24S/F297Y) expression and cell autolysis induced with lactose in HCDC. (DOCX 3264 kb)

## Abbreviations

CBM: Cellulose-binding module
HCDC: High cell density culture
IPTG: Isopropyl-β-D-1-thiogalactopyranoside
*p*NPG: *p*-Nitrophenyl β-D-glucopyranoside
