# Supplementary material for: Light induced expression of β-glucosidase in Escherichia coli with autolysis of cell
Source: BMC Biotechnol. 2017 Nov 7;17:74. doi: 10.1186/s12896-017-0402-1 (PMC5688802; doi:10.1186/s12896-017-0402-1)
Supplement: Additional file 1: Table S1. — Bacterial strains and plasmids used in this study. Table S2. Oligo nucleotides used in this work. Table S3. Expression of prokaryotic β-glucosidase. Figure S1. SDS-PAGE of Bgl1A(A24S/F297Y) expression and cell autolysis induced with lactose in HCDC. (DOCX 3264 kb) [file 12896_2017_402_MOESM1_ESM.docx]

**Light induced** **expression of β-glucosidase in** ***Escherichia coli*** **with auto****lysis of cell**

**Fei Chang^1,2,3,#^, Xianbing Zhang^1,2,3,#^, Yu Pan^1^, Youxue Lu^1^, Wei Fang^1,2^, Zemin Fang^1,2,3,*^, Yazhong Xiao^1,2,3,*^**

^1^ School of Life Sciences, Anhui University, Hefei, Anhui 230601, China

^2^ Anhui Key Laboratory of Modern Biomanufacturing, Hefei, Anhui 230601, China

^3^ Anhui Provincial Engineering Technology Research Center of Microorganisms and Biocatalysis, Hefei, Anhui 230601, China

^#^ these authors contributed equally to this work.

* Correspondence

Phone/Fax: +86 551 63861861

E-mail: zemin_fang@ahu.edu.cn (to Z. Fang); yzxiao@ahu.edu.cn (to Y. Xiao).

**Table S1**. Bacterial strains and plasmids used in this study

| Strains/plasmids | Relevant genotype and characteristics | Reference | |
| --- | --- | --- | --- |
| Strains | | | |
| *E. coli* Trans5α | F^-^, Φ80 *lac*ZΔM15 Δ(*lac*ZYA-*arg*F) U169 *end*A1 *rec*A1 *hsd*R17(rK^-^,mK^+^) *sup*E44λ^-^ *thi* -1 *gyr*A96 *rel*A1 *pho*A | | TransGen Biotech (Beijing, China) |
| *E. coli* BL21 (DE3) | F^−^, *ompT*, *hsdS*(rB^−^ mB^−^), *gal*, *dcm*(DE3) | | TransGen Biotech (Beijing, China) |
| *E. coli* BL21 (DE3)pLysS | F^−^, *ompT*, *hsdS*(rB^−^ mB^−^), *gal*, *dcm*(DE3) pLysS (Cm^R^) | | TransGen Biotech (Beijing, China) |
| Plasmids | | | |
| pET-22b-T7-*bgl* | pET-22b(+) derivative carrying *bgl**1A(A24S/F297Y)* fragment with T7 *lac* promoter, Ap^R^ | | Fang et al. 2016 |
| pUC57-*pD* | pUC57 derivative carrying *pD* synthetic fragment, Ap^R^ | | This study |
| pUC57-*SRRz* | pUC57 derivative carrying *S**RRz* synthetic fragment, Ap^R^ | | This study |
| pUC57-*CBM* | pUC57 derivative carrying *CBM* synthetic fragment, Ap^R^ | | This study |
| pET-22b-pD-*bgl-CBM*-T7-*SRRz* | pET-22b-pD derivative carrying *bgl-CBM* fragment(induced by light) and SRRz fragment initiated by the T7 promoter, Ap^R^ | | This study |

**Table S2**. Oligo nucleotides used in this work

| Oligo muclcotides | Sequence(5’-3’) | Restriction enzyme | Target sequence |
| --- | --- | --- | --- |
| pD-F | TATT*TCTAGA*GCAACCATTATCACCGCCA | *Xba* I | *pD* |
| pD-R | GCGGC*GACGATAGTC*ATGCCCACTTACATCAATTGTCA | *Psh*A I |  |
| bgl-F | TATA*CATATG*ACTAAAATATCTTTACCAACTTGTT | *Nde* I | *bgl1A(A24S/F297Y)* |
| bgl-R | GGTG*CTCGAG*TTATCTATTTGAGATTAATGCTTTATAGG | *Xho* I |  |
| pD-bgl-F | GCGGC*GACGATAGTC*ATGCCCACTTACATCAATTGTCA | *Psh*A I | *pD-bgl1A(A24S/F297Y)* |
| pD-bgl-R | ggggcat*gactatcgtc*atccggatatagttcctcctttcag | *Psh*A I |  |
| SRRz-F | TATA*CATATG*ATGAAGATGCCAGAAAAACATGA | *Nde* I | *SRRz* |
| SRRz-R | GGTG*CTCGAG*CTATCTGCACTGCTCATTAATATACTTCT | *Xho* I |  |
| Bgl-CBM-R | TCTATTTGAGATTAATGCTTTATAGG |  | *bgl1A(A24S/F297Y)* |
| CBM-F | CCTATAAAGCATTAATCTCAAATAGAGGCTCCGCTGGCTCCGCTATGGATCTGAAAGTTGAATTTTACA |  | *cbm* |
| CBM-R | GGTG*CTCGAG*ACCCGGTTCTTTACCCCAA | *Xho* I |  |

**Table S3** Expression of prokaryotic β-glucosidase

| Name | inducer | Cell disrupt method | Protein purification method | yield | Refs |
| --- | --- | --- | --- | --- | --- |
| BglA | IPTG | Sonication | Hexahistidine affinity | 0.531 U/mL | [1] |
| BglPm | IPTG | Sonication | GST affinity | n.r. | [2] |
| Unbgl1A | IPTG | Sonication | Hexahistidine affinity | n.r. | [3] |
| O08324 | IPTG | Sonication | Hexahistidine affinity | n.r. | [4] |
| BGL | IPTG | Extracellular expression | Hexahistidine affinity | 1.67 U/mL | [5] |
| MeBglD2 | Autoinduction medium (Novagen) | BugBuster (Novagen) | Hexahistidine affinity | n.r. | [6] |
| β-glucosidase | IPTG or lactose | Sonication | Anion exchange chromatography | 238 mg/L | [7] |
| *Ho*BGLA | IPTG | Homogenization | Hexahistidine affinity | 1.5 mg/L | [8] |
| SC7558 | IPTG | Sonication | Hexahistidine affinity | 2,650 U/mL | [9] |
| Bgl1A(A24S/F297Y) | Light | autolysis | CBM affinity | 83.15 U/mL | this study |

n.r. = not reported.

**Figure S1** SDS-PAGE of Bgl1A(A24S/F297Y) expression and cell autolysis induced with lactose in HCDC. The sample of 16 h post-induced with lactose was incubated in 4 ^o^C and the lysis efficiency was detected every 4 h. Lysis reflect supernatant of fermentation culture after cell autolysis, super and precip reflect supernatant and precipitate after ultrasonic fragmentation of cells that were not autolysed.

**
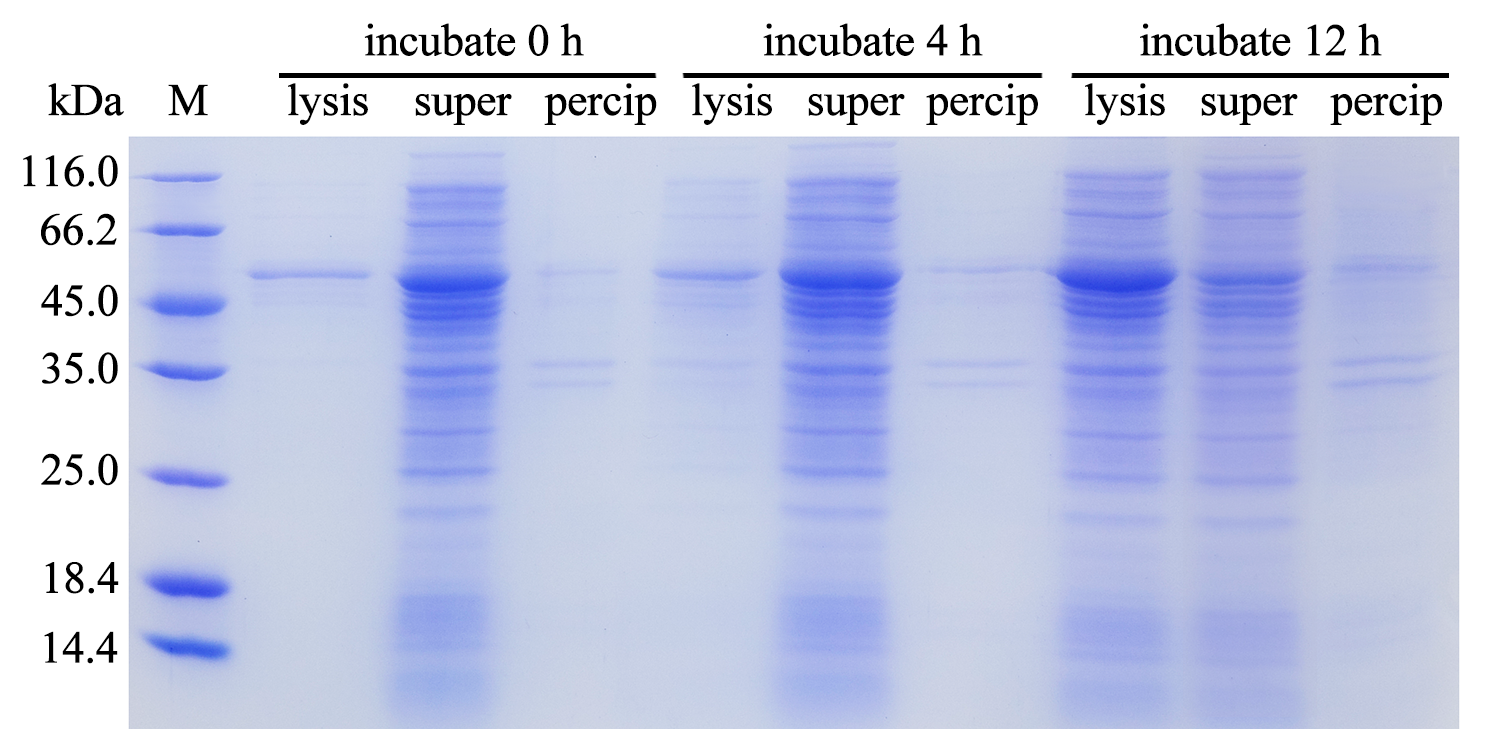
**

**References**

1. Chang J, Park IH, Lee YS, Ahn SC, Zhou Y, Choi YL. Cloning, expression, and characterization of β-glucosidase from *Exiguobacterium* sp. DAU5 and transglycosylation activity. Biotechnol Bioprocess Eng. 2011;16(1):97-106.

2. Cui CH, Kim JK, Kim SC, Im WT. Characterization of a ginsenoside-transforming β-glucosidase from *Paenibacillus mucilaginosus* and its application for enhanced production of minor ginsenoside F_2_. PLoS One. 2014;9(1).

3. Lu J, Du L, Wei Y, Hu Y, Huang R. Expression and characterization of a novel highly glucose-tolerant β-glucosidase from a soil metagenome. Acta Biochim Biophys Sin (Shanghai). 2013;45(8):664-73.

4. Sinha SK, Datta S. β-Glucosidase from the hyperthermophilic archaeon *Thermococcus* sp. is a salt-tolerant enzyme that is stabilized by its reaction product glucose. Appl Microbiol Biotechnol. 2016;100(19):8399-409.

5. Chamoli S, Kumar P, Navani NK, Verma AK. Secretory expression, characterization and docking study of glucose-tolerant β-glucosidase from *B. subtilis*. Int J Biol Macromol. 2016;85:425-33.

6. Matsuzawa T, Yaoi K. Screening, identification, and characterization of a novel saccharide-stimulated β-glycosidase from a soil metagenomic library. Appl Microbiol Biotechnol. 2017;101(2):633-46.

7. Naz S, Ikram N, Rajoka MI, Sadaf S, Akhtar MW. Enhanced production and characterization of a β-glucosidase from Bacillus halodurans expressed in *Escherichia coli*. Biochemistry (Mosc). 2010;75(4):513-18.

8. Hassan N, Thu-Ha N, Intanon M, Kori LD, Patel BKC, Haltrich D, et al. Biochemical and structural characterization of a thermostable β-glucosidase from *Halothermothrix orenii* for galacto-oligosaccharide synthesis. Appl Microbiol Biotechnol. 2015;99(4):1731-44.

9. Gu MZ, Wang JC, Liu WB, Zhou Y, Ye BC. Expression and displaying of β-glucosidase from *Streptomyces coelicolor* A3 in *Escherichia coli*. Appl Biochem Biotechnol. 2013;170(7):1713-23.
